# Transformation of Silicate Ions into Silica under the Influence of Acid on the Structure of Serpentinite

**DOI:** 10.3390/molecules29112502

**Published:** 2024-05-25

**Authors:** Abdrazak Auyeshov, Kazhymuhan Arynov, Chaizada Yeskibayeva, Aizhan Dikanbayeva, Darkhan Auyeshov, Yerkebulan Raiymbekov

**Affiliations:** 1 High School of Chemical Engineering and Biotechnology, M. Auezov South Kazakhstan University, Shymkent 160000, Kazakhstan; yeskibayeva@internet.ru (C.Y.); dikanbaeva86@mail.ru (A.D.); centersapa@mail.ru (D.A.); eplusr@bk.ru (Y.R.); 2 “Aspan Tau LTD” LLP, Almaty 050006, Kazakhstan; tau_aspan@mail.ru

**Keywords:** serpentinite, IR spectroscopy, layered silicate, silica, sulfuric acid

## Abstract

The process of transformation of the silicate components of the crystal lattice structure of chrysotile during its quantitative interaction with aqueous solutions containing various stoichiometrically required amounts of sulfuric acid (SRA H_2_SO_4_) calculated with respect to the magnesium content in the composition of chrysotile is investigated. It has been shown by IR spectroscopic, X-ray phase, thermal and chemical methods of investigation and analysis that, with quantitative interactions of chrysotile and sulfuric acid, first of all, the “brucite layer” of the molecular structural structure of chrysotile is exposed to acid at SRA H_2_SO_4_ = 0.1–0.3. As a result of ion exchange processes, acidic silanol (≡Si–O–H) or disilanol (=Si=(O–H)_2_) bonds are formed. These acid groups form one-dimensional silicate chains with transverse bridges (≡Si–O–Si≡), where the angles (Si–O–Si = 180 °C) straighten, which are recorded in the IR spectra in the region of characteristic absorption of 1220–1250 cm^−1^ silica. The association of the resulting acid groups into silicate chains, dimers, and trimers with transverse bridges, leads to the appearance of colloidal silica particles in the system, which cause some inhibition of the dissolution of layered magnesium hydrosilicate in sulfuric acid solutions containing H_2_SO_4_ ˃ 0.3 SRA.

## 1. Introduction

Crushed serpentinites are one of the few minerals rich in magnesium and silica content (wt.% MgO to 43, SiO_2_ to 45). As a result, they have been the object of numerous scientific studies aimed at extracting from them in the form of metallic magnesium [1], magnesium salts [2,3], hydroxide [4,5] and magnesium oxide [6], as well as silica [7,8]. However, to date, the technology for obtaining the above-mentioned magnesium compounds based on the processing of serpentinite by acidic methods has not been widely used in practice.

One of the known main reasons for the lack of technology for processing magnesium hydrosilicates, in particular serpentinites, based on acidic methods of their processing, is the occurrence of colloidal particles or gels of silicic acids during the leaching of serpentine. The consequence of this phenomenon leads to a decrease in both the rate of further dissolution of serpentine in aqueous solutions of mineral acids and the complication of the technological process of separation of a productive magnesium solution, that is, filtration. For this reason, in our opinion, acidic methods of processing both natural hydrosilicates and serpentinite (layered magnesium hydrosilicates) waste from mining companies of the chrysotile mining industries are not widely used in practice. In the scientific literature, especially in patents [9] intended for “methods of processing magnesium hydrosilicates or serpentinite”, this circumstance is often superficially paid attention to due to little or no scientific information based on the results of data from fundamental studies of this phenomenon and circumstance. The kinetics of the dissolution of serpentinite in acids are generally controlled by internal diffusion through the product layer of the dissolution of the silicate component of magnesium hydrosilicate, as indicated by the results of studies conducted in recent years [10,11]. Therefore, a deeper study requires the course of changes occurring in the silicate part in the molecular structure of serpentine during its interactions with acid solutions, that is, the transformation processes of the silicate component into various forms of silicic acid and products of its associations—colloidal particles.

In this article, the transformation of the silicate component in the molecular structure of chrysotile [Mg_3_Si_2_O_5_(OH)_4_] (the most famous mineral from the serpentine group) into silica is investigated by quantitative interaction with solutions of various molar concentrations of sulfuric acid by the IR-Fourier spectroscopic method, since the products of silicon dissolution, in this case amorphous silica, are anX-ray amorphous substance.

Identification of the processes of occurrence of colloidal particles or gels of silicic acid (amorphous silica) in the system was carried out based on studying the IR spectra of acid-insoluble residues obtained when used to dissolve chrysotile in aqueous solutions containing various stoichiometrically required amounts of sulfuric acid (SRA H_2_SO_4_).

The research results can be useful in the development of new schemes for the processing of magnesium hydrosilicates, including serpentinite and serpentinite waste from the processing of chrysotile ore using acidic methods, as well as new materials and methods for carbon capture and storage (CCS) based on serpentine.

## 2. Results

**Figure 1:**
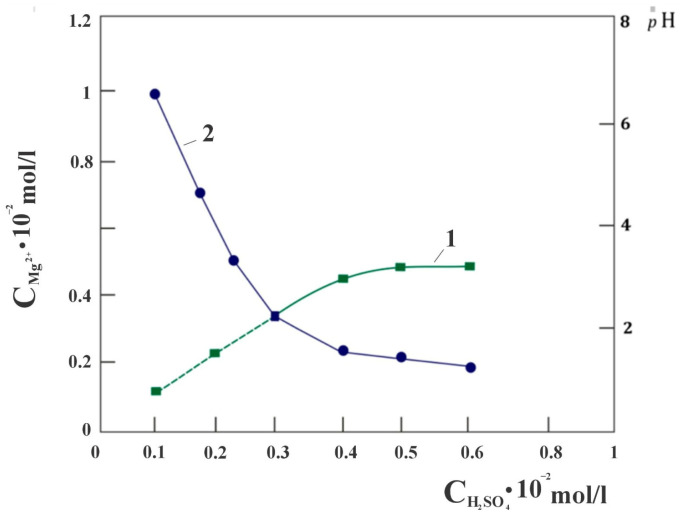
Dependence of the degree of magnesium extraction (1) and pH of the solution (2) on the SRA of sulfuric acid.

**Figure 2:**
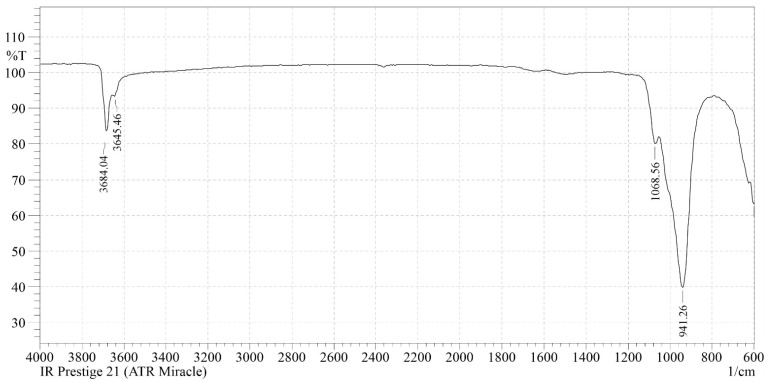
The IR spectrum of the initial chrysotile.

When studying the quantitative interaction of chrysotile and sulfuric acid, attention was drawn to the fact that the dissolution of chrysotile proceeds with the extraction of magnesium into a sulfate solution, in almost an equivalent amount in the concentration range of the stoichiometrically required amount of sulfuric acid from 0 to 0.3 SRA H_2_SO_4_ in the solution used (Figure 1), calculated with respect to the molar content of magnesium in the sample chrysotile. Further, the proportional extraction of magnesium into the solution from SRA H_2_SO_4_ gradually becomes impaired. It was assumed that this phenomenon may be due to the presence of free brucite in the composition of chrysotile taken for the study. In order to verify the absence of free Mg(OH)_2_ brucite in the composition of chrysotile, a derivatogram of the studied chrysotile sample, pre-baked at a temperature of 500 °C, was removed (Figure 2). As can be seen, there is no peak characteristic of the decomposition of brucite at a temperature of 350–450 °C with a maximum of 400 °C. This means that the acid, upon dissolution of the mineral, interacts only with the molecular structure of serpentine [Mg_3_Si_2_O_5_(OH)_4_].

The discovered nature of the interaction of chrysotile and acid was the reason for the hypothesis that magnesium, located in various fragments of the structural and molecular structure of chrysotile, dissolves under different conditions during the leaching of chrysotile.

From the idelized structure of the crystal lattice of chrysotile [12], the two-layer packages composing the structure are characterized by the disproportionality of trioctahedral “brusite” layers with hexagonal grids of silicon-oxygen tetrahedra, which causes an uneven distribution of magnesium ions in individual packages of layered hydrosilicate. Based on this structure, the structural and molecular formula of chrysotile can be depicted more clearly: Mg_3_Si_2_O_5_(OH)_4_ = Mg(OH)_2_–(MgOH)_2_–Si_2_O_5_ with the assumption that 1/3 of the magnesium, mainly in the form of—[Mg(OH)_2_], is in the “brucite” octahedral layer, and the other 2/3 part in the form of—(MgOH)_2_ are arranged in a tetrahedral.

However, both forms, despite the structurally different environment of magnesium ions by the silicate components of the chrysotile molecule, may be equally accessible to the effects of H_3_O^+^ ions, but may have different kinetic characteristics of leaching into solution. Under this assumption, the found equivalent amount of magnesium ions in solution when using an equivalent amount of acid up to 0.3 SRA H_2_SO_4_ has its logical explanation; however, the reasons for some inhibition of the process with a further increase in the amount of SRA H_2_SO_4_ lead to a more detailed study. It is known that the dissolution of a mineral solid proceeds through two different processes: the first is a rapid reaction between surface areas and reagents, mainly H_3_O^+^, OH^−^ or ligands, leading to the formation of surface particles, followed by a slower phase when the metal separates from the surface and passes into solution. In this case of the chrysotile mineral, rapid mass transfer is initially observed due to proton exchange with available magnesium cations of the “brucite layer” on the surface of the chrysotile fibers. The rapid change in the pH of the suspension observed at the initial stage of the interaction “chrysotile—SRA H_2_SO_4_” reflects the fact that the interaction has an acid-base character (Figure 1, curve 2). A sharp change in the pH of the suspension after the point of intersection of the lines of dependence of the amount of magnesium (C_Mg_^2+^) in solution on the SRA H_2_SO_4_ also confirms the transition of the dissolution process to another kinetic regime. From the results of leaching chrysotile with sulfuric acid solutions, it can also be noted that with an SRA of H_2_SO_4_ ˃ 0.4, the filtration rate of the suspension decreases sharply, from υ = 28·10^−2^ mL/min·cm^2^ to υ = 16.2·10^−2^ mL/min·cm^2^. It was obvious that one of the main reasons for the observed violations of the linear dependencies of C_Mg_^2+^—SRAH_2_SO_4_ and pH—SRAH_2_SO_4_ when using SRA H_2_SO_4_ ˃ 0.3 in solution is the occurrence of a silicon-containing product in the system, leading to some inhibition of the chrysotile dissolution process. Further, we studied this phenomenon using the possibility of IR Fourier spectroscopy, since the probable products of silicon dissolution are X-ray amorphous substances. The study was carried out by removing the Fourier infrared spectra of acid-insoluble residues after each leaching of chrysotile with sulfuric acid solutions containing different amounts of SRA H_2_SO_4_, at constant values of the volume and temperature of the solution.

In the IR spectrum of the initial chrysotile fiber (Figure 2), in the high-frequency region, the doublet of narrow bands at ν_asOH_ = 3684 cm^−1^ and ν_asOH_ = 3645 cm^−1^ are asymmetric, ν_sOH_ = 2931 cm^−1^ are symmetric valence vibrations of the OH group of tetrahedral silica [13,14]. The oscillation at ν_asOH_ = 3684 cm^−1^ refers to the intraglobularpart, and ν_asOH_ = 3645 cm^−1^ to the surface part of the structure of themineral [14,15,16]. The absence of a narrow band ν_asOH_ = 3750 cm^−1^ with its deformation component at δ_OH_ = 870 cm^−1^ indicates that there are no free or isolated surface OH groups in the tetrahedral silica of chrysotile. The deformation oscillation of the hydroxyl group δ(OH) of the adsorption water is recorded at δ(H_2_O) = 1640 cm^−1^ [14,15,16]. Two deep and narrow absorption bands at 941 cm^−1^ with a shoulder at 1064 cm^−1^ are characteristic of layered silicates for groups (SiO_4_) [15,17]. The absorption band 941 cm^−1^ refers to the valence asymmetric vibrations of the ν_as_ = O–Si–O(Mg) bond, the valence vibration of tetrahedral silica perturbed by an octahedral brucite-like layer. The shoulder at 1064 cm^−1^ is a valence asymmetric oscillation of ν_as_ = Si–O–Si tetrahedral silica. The oscillation in the low–frequency region at 601, 551 and SiO_4_—455 cm^−1^ refers to the δ-deformation vibrations of the SiO_4_ tetrahedron [16,17].

**Figure 3:**
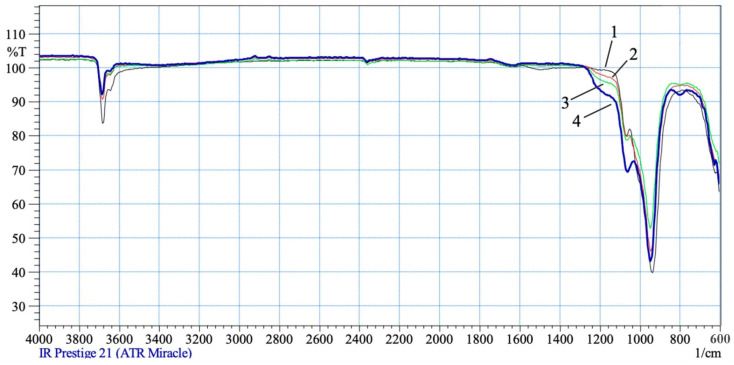
IR spectra of the original chrysotile treated with solutions of 0.1–0.5 SRA H_2_SO_4_: 1—0.1 SRA H_2_SO_4_; 2—0.2 SRA H_2_SO_4_; 3—0.3 SRA H_2_SO_4_; 4—0.5 SRA H_2_SO_4_.

In the IR spectra of acid-insoluble chrysotile residues treated with 0.1 and 0.2 SRA H_2_SO_4_, no significant changes are observed in the range of OH group fluctuations (Figure 3: line-2). In the spectrum of chrysotile treated with 0.3–0.5 SRA H_2_SO_4_, the intraglobular peak of hydroxyl ν_asOH_ = 3684 cm^−1^ and the peak of hydroxyl of the surface part of the mineral structure at ν_asOH_ = 3645 cm^−1^ is slightly reduced in intensity. On the threshold of the valence fluctuations ν_as_(Si–O–Si) in the area, 1120–1250 cm^−1^ appears a wide “step” of absorption, which becomes more intense with the rise of the SRA H_2_SO_4_ from 0.3 to 0.5. The appearance of this wide “step” of absorption indicates the presence and concentration of various acid groups of silica (Si–O–Si–ON^+^) on the surface of the chrysotile fibers [14,15,16,17]. As the SRA H_2_SO_4_ increases from 0.3 to 0.5 and the magnesium dissolves, the integrity destructions in the crystal lattice of the tetrahedral–octahedral layered pair increase, resulting in silica debris, di-dextras, tri-, tetro-, pentomers, etc., which interact with hydrogen ions and are associated with each other to varying degrees. Each hydrogenated silica fragment is recorded at an individual peak in the 1120–1250 cm^−1^ IR region, overlapping with each other to form a wide “step” of absorption. At the same time, the asymmetric valence oscillation ν_as_ = O–Si–O(Mg) at 941 cm^−1^ loses a slight intensity, due to the dissolution of magnesium from the brusitoid-like octahedral layer, and the silica arm ν_as_(Si–O–Si) = 1064 cm^−1^ begins to stand out with a pronounced peak, its symmetrical valence oscillation appears at ν_as_(Si–O–Si) = 802 cm^−1^ [14,15,16,17].

**Figure 4:**
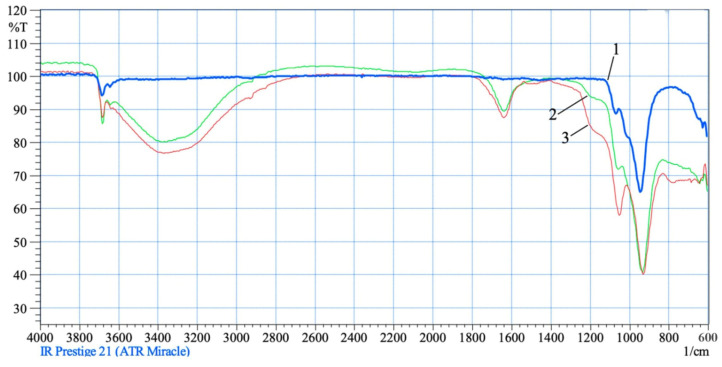
IR spectra of untreated (1) and acid-treated (2—0.5 SRA H_2_SO_4_; 3—0.8 SRA SRA H_2_SO_4_) chrysotile.

The observed phenomena are enhanced by the interaction of chrysotile with solutions of 0.6–1.0 SRA H_2_SO_4_ (Figure 4). In the IR spectra of acid-treated chrysotile residues, the intraglobular peak intensity of hydroxyl ν_asON_ = 3684 cm^−1^ increases slightly, but as before, it does not change its position, and the peak of hydroxyl ν_asON_ = 3645 cm^−1^ characterizing the surface part of chrysotile significantly increases its intensity and is recorded in the spectrum range from 3620 cm^−1^ to 2840 cm^−1^ wide and with an intense “hump”. The deformation oscillation of water also increases intensity and is recorded as a peak of average intensity at δ(H-ON) = 1640 cm^−1^. The increase in the intensity and expansion of the absorption area of the silica acid group (Si–O–Si–ON^+^) in the 1120–1240 cm^−1^ region with an increase in the SRA H_2_SO_4_ 0.6–1.0 indicates an increase in the destruction of the integrity of the tetrahedral–octahedral layer of a crystal lattice pair of a chrysotile structure, as well asan increase in the varieties of silica “fragments” and in their degree of hydration and association. All the fragments of silica acid grouping (Si–O–Si)–ON^+^ recorded by an individual peak in one IR region, further intensify the intensity and extend the absorption area from 1120 cm^−1^ to 1240 cm^−1^. Asymmetric oscillations of hydroxyl—ν_asOH_, in the composition of silica “fragments” are recorded by individual peaks in the high-frequency spectrum, but overlap, increasing the total intensity of the OH bond in the broad spectrum from 3620 cm^−1^ to 2840 cm^−1^ (large and wide “hump”).

**Figure 5:**
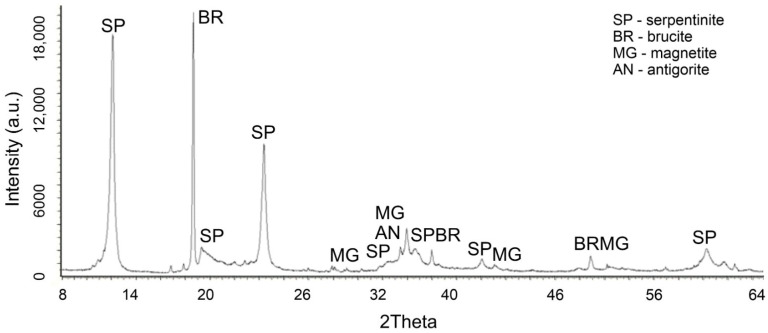
XRD of the initial serpentinite.

All observed changes at 0.3–1.0 H_2_SO_4_ SRA are significantly amplified, which indicates an increase in the concentration of various HE-related acid groups on the surface of the structural structure of thechrysotile. It should be noted that the oscillation intensity at 941 cm^−1^ does not decrease, but increases, possibly as a result of the application of the valence oscillation of the acid hydroxyl group Si–OH^+^ with the valence oscillation ν_as_ = O–Si–O(Mg). Shoulder—the silica absorption band ν_as_(Si–O–Si) = 1064 cm^−1^ is separated by a separate peak. The observed phenomena, in sum, can testify to the strength of destruction of the integrity of the structure of a tetrahedral–octahedral layered pair and about the appearance on a surface of a globule of acid hydroxyl group Si–ON^+^, in a system-independent phase of silica formed. The absorption bands manifested in the IR spectra, characterizing the transformations of the silicate components of chrysotile molecules into silica, are more strongly expressed in the IR spectra of residues obtained by treating chrysotile with solutions of 70% (5) and 100% (6) SRA H_2_SO_4_ (Figure 5). In the IR spectra of chrysotile residues (5 and 6), the intraglobular peak ν_asOH_ = 3684 cm^−1^ does not change as before, wide and deep peaks of acid groups appear in the range from 3620 to 2840 cm^−1^. At the same time, the deformation vibrations of the water also deepen and are recorded by a peak of average intensity at δ(SiOH^+^) = 1640 cm^−1^. The absorption step at the threshold of the valence vibrations of silica at δ(SiOH^+^) = 1120–1250 cm^−1^ is recorded by a deep hump. Both uptakes are much more intense than in the previous residues. Silica peak at ν_as_(Si–O–Si) = 1060 cm^−1^ continues to increase in intensity. All observed changes deepen significantly, which indicates an increase in the concentration of various OH^+^-associated acid groups on the surface of chrysotile molecules. The area of deformation fluctuations of chrysotile is below 800 cm^−1^, and it can be said that it is significantly destroyed.

Tracing the course of changes in the Fourier-infrared spectra of acid-insoluble residues obtained after treatment of chrysotile with solutions containing from 0.1 to 1.0 SRA H_2_SO_4_ shows that the appearance of silica-forming products becomes noticeable when using solutions containing 0.3–0.4 SRA H_2_SO_4_, which can then be associated in an acidic environment into larger colloidal particles. The transformation of the silicate components of the chrysotile molecule into silica apparently begins with the destruction of the “brucite layers” of the molecular structure of chrysotile.

**Figure 6:**
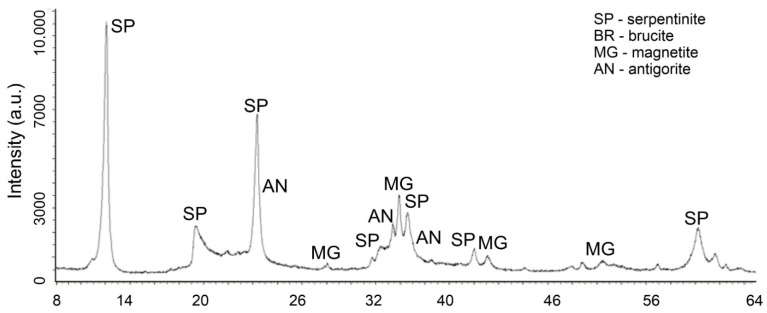
XRD of serpentinite treated with 40% SRA H_2_SO_4_ (1 time washed with water) after treatment.

In favor of this statement, changes in the X-ray of the initial chrysotile (Figure 5) and the acid-insoluble residue (Figure 6) obtained after treatment with a solution of 0.4 SRA H_2_SO_4_ are given.

Changes in the phase composition of serpentinite after treatment with solutions of 0.1–1.0 SRA H_2_SO_4_ were traced by X-ray phase analysis. A comparative analysis of X-ray images (Figure 5) of the initial serpentinite and the same serpentinite after treatment with 0.4 of the SRA with H_2_SO_4_ solution (Figure 6) shows that the prescribed peaks to serpentinite (chrysotile and antigorite) Mg_3_Si_2_O_5_(OH)_4_ at values for chrysotile d/n = 7.38–4.619–3.661–2.487–2.141–1.53 Å and antigorite d/n = 7.30–3.63–2.51 Å do not undergo significant changes after acid treatment [18,19,20,21].

The XRD (Figure 6) lacks peaks belonging to brucite, marked in Figure 6 with peaks at d/n = 4.77–2.365–1.794 Å. The remaining peaks found in the initial serpentinite are chrysotile d/n = 7.38–4.619–3.661–2.487–2.141–1.53 Å and antigorite d/n = 7.30–3.63–2.51 Å and magnetite—Fe[Fe_2_O_3_] (d/n = 2.99–2.541–2.098–1.710–1.612 Å), when treated with H_2_SO_4_ solutions, up to 0.4 of the SRA practically do not undergo any special changes. These data show that complete destruction of the structural structure of the silicate component of serpentinite does not occur.

## 3. Discussion

Starting to discuss the results of IR-Fourier spectroscopy, we pay attention to the general, interrelated patterns of changes in the IR spectra of chrysotile samples treated with acid. In the IR spectra of chrysotile samples in the area of acid concentration taken (0.3–0.7) of SRA H_2_SO_4_, there are progressive strong and wide bands in the frequency ranges 3620–3000 cm^−1^, 1640 cm^−1^ and 1250–1120 cm^−1^ (Figure 4). The 3620–3000 cm^−1^ region is characteristic of asymmetric vs and symmetric vs valence oscillations of adsorbed OH [14,15]. The location, type and intensity of this oscillation depend on the nature of the relationship of the water molecule with the structure of the mineral. The location, type and intensity of this oscillation depends on the nature of the relationship betweenthe water molecule with the structure of the mineral. The weaker the bond between OH and the crystal structure, the higher the frequency of water vibrations; the stronger the bond, the lower the frequency of vibrations. Figure 4 shows that the initial chrysotile has two small sharp peaks at 3684 cm^−1^ and 3650 cm^−1^, which belong to ν_as_ and ν_s_ weakly bound OH. At concentrations of 0.3–1.0 H_2_SO_4_ SNC, the peak at 3684 cm^−1^ remains in place, and the peak of 3650 cm^−1^ increases intensity and expands into the low-frequency region, forming a strong and broad uptake from 3620–3000 cm^−1^. A small sharp peak at 3684 cm^−1^, which is not affected in all treated chrysotile samples with acid, suggests the presence of an isolated OH^−^ ion or weakly bound to the structure of the water crystal. The high peak frequency may be due to the parallel orientation of the H-H vector of the crystal axis [14,15].

The increase in intensity and the expansion of the peak of 3650 cm^−1^ towards the low-frequency region, forming strong and wide bands of 3620–3000 cm^−1^ can be explained by the change in texture of the structure of the tetrahedral-octahedral layer of the pair of the structure of chrysotile.

**Figure 7:**
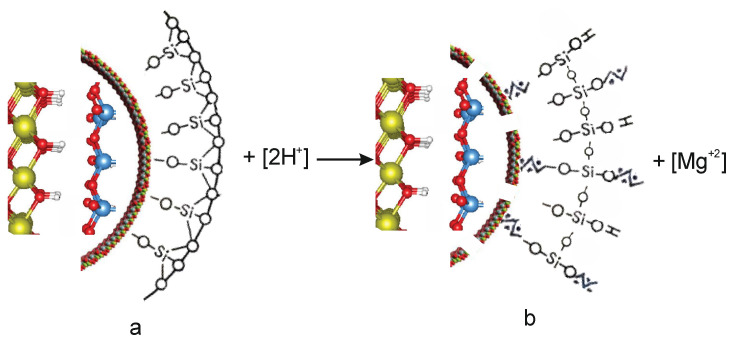
The globule model of the initial (**a**) and acid-treated (**b**)chrysotile.

As is known, the structural unit of serpentinites is a 0.72 nm thick layer in which a trioctahedral sheet Mg[Mg(OH)_4_]_6_ is connected to a tetrahedral sheet [Si_2_O_5_]_2_, forming a tetrahedral-octahedral layered pair (T-O), which, on the other hand, are connected to the following (T-O) layers weak H-bond [12,13]. Antigorite has lamellar or scaly forms, lizardite has a flat multilayer crystal structure, and chrysotile has a tubular, convoluted structure, an inner octohedral [Mg(OH)_2_], an outer tetrahedral [Si_2_O_4_] layer (Figure 7) [12,13].

When chrysotile and H_2_SO_4_ interact, the integrity of the tetrahedral-octahedral layered pair is destroyed, which affects the structure of chrysotile to varying degrees, depending on the quantitative ratio of magnesium in serpentinite and sulfuric acid [Chr(Mg) ÷ H_2_SO_4_].

When exposed to H_2_SO_4_ concentrations of 0.3–1.0 SRA H_2_SO_4_, an acid-base interaction occurs between the octahedral layer of chrysotile, in which part of the magnesium leaches into the solution. At the same time, hydrogen ions H^+^ are adsorbed onto the tetrahedral silica layer, and acidic Si–OH^+^ hydroxyl groups appear on the surface of the tetrahedral silica layer (Figure 7). The absorption band 930 cm^−1^ belongs to the valence vibrations of the acid hydroxyl group Si–OH^+^, where hydroxyl fluctuates as a single mass [13,14,15]. In the IR spectra of serpentinites, this band is superimposed with a band of 941 cm^−1^ ν_as_ = O–Si–O(Mg); therefore, it is not possible to detect it separately, but the intensity of this peak decreases at a concentration below 0.3 SRA H_2_SO_4_; due to the destruction of surface ν_as_ = O–Si–O(Mg) bonds above 0.3 SRA H_2_SO_4_, the intensity of this band begins to increase, as above 0.3 SRA the concentration of acid hydroxyl group Si–OH^+^ growing. In the high-frequency region of the IR spectra, the acid OH^+^ group is spelled at 3630 cm^−1^;with the growth of the SRA H_2_SO_4,_this band gradually increases the intensity and expands, which indicates an increase in the concentration of the OH^+^ group and an increase in their diversity.

**Figure 8:**
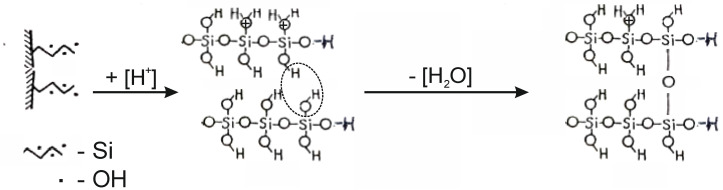
The mechanism of formation of siloxane bonds < Si–O–Si = 180 °C when exposed to acid on the tetrahedral layer of the chrysotile molecule package.

**Figure 9:**
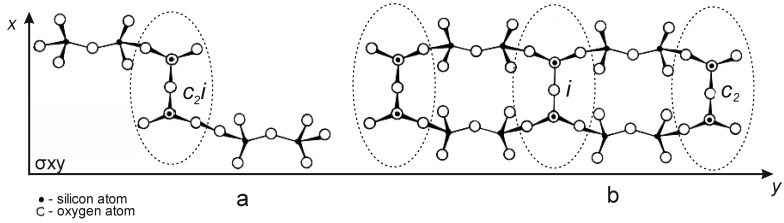
Diagram of the structure of condensed chains (**a**) and ribbons (**b**) of tetrahedral SiO_4_ bonded by bridged siloxane bonds < Si–O–Si = 180 °C.

In parallel with the adsorption of hydrogen ions H^+^, water molecules and hydroxonium H_3_O^+^ ions are adsorbed onto the surface of tetrahedral silica. The integrity of the tetrahedral–octahedral layered pair is destroyed. A wide range of “fragments” appear on the surface of silica: dimers, trimers, oligomers, chains, ribbons of silica from SiO_4_ tetrahedra interconnected by Si–O–Si bridging bonds, the angles between which are 180° (Figure 8) [15]. The appearance of a wide adsorption band in the IR spectrum at ~1200 cm^−1^ indicates the existence of Si–O–Si angles close to 180° in a complex silicate anion (Figure 9a). This band is most characteristic of silicates containing ribbons and layers. A special feature of these tapes is the transverse bridges straightened to <Si–O–Si = 180°. The frequency of ~1200 cm^−1^ belongs to the asymmetric oscillation of the rectified siloxane group ν_as_(Si–O–Si), where the oxygen atom plays the role of a bridge between two silicate chains, between tetrahedral SiO_4_ groups (Figure 9b).

The appearance of such a wide “band” of absorption of OH^+^ communication in the IR 3620–3000 cm^−1^ area is also explained by the increase in anharmonicity of the oscillation associated with the diversity of the force of the O–H^+^ bond with mineral fragments, numerous neoplasms: dimers, trimmers, tetramers and other “fragments” of silica. In addition, the pH range = 1–4 is very favorable for the formation of H-linked complexes of silicic acid, hydrogen-bound or associated siloxane molecules, polysilic acids having a layered structure with interlayer H-linked hydroxyls, which are characterized by a wide absorption band of 3200–3300 cm^−1^. The increase not only of the width, but also of the intensity of the IR spectrum in the region of ~1200 cm^−1^ and 3620–3000 cm^−1^ with the increase of the SRA of acid above 0.5 SRA testifies to the depth of the changes taking place in the structure of the octahedral–tetrahedral pair of chrysotile, and to the thickness of the “fragments” layering of the reaction on the surface of the globule.

At a concentration of sulfuric acid (0.7–1.0) SRA, the degree of the extracted amount of magnesium in the solution does not exceed 0.45–0.7 of its total amount in the composition of chrysotile. Apparently, at these concentrations of sulfuric acid in solution, as a result of the transformations considered, a sufficiently dense layer of chain silicate colloidal particles is formed on the surface of the fibers, leading to inhibition of the process of dissolution of chrysotile in acid solutions.

In all IR spectra of chrysotile treated with acid, the main bands of chrysotile, a small sharp peak ν_asOH_ = 3684 cm^−1^, an intense peak of the bond ν_as_(O–Si–O) = 948 cm^−1^ with a shoulder of tetrahedral silica ν_as_(Si–O–Si) = 1068 cm^−1^ continue to be present. Maintaining its position without changing the data of the three main peaks of chrysotile absorption when treated with sulfuric acid up to 1.0 SRA H_2_SO_4_ indicates that in a chrysotile microparticle, all changes affect only the surface or peripheral part of it, when the internal volume of the particle remains intact.

## 4. Materials and Methods

A sample of chrysotile (Chr) (grade A-4-20, Kostanay Minerals JSC, Zhitikara city, Kazakhstan) in the amount of 20 g was crushed and sieved, from which a particle fraction of <0.14 mm in size, weighing 10.0 g containing, by weight, %: 26.42 Mg; 18.8 Si; 2.7 Fe, 0.21 Cr, 0.17 Mn, 0.53 Ca, 0.48 Al. The amount of magnesium and iron in 10.0 g (Chr) was 0.11 mol and 0.005 mol, respectively.

The stoichiometric required amount (SRA) of sulfuric acid for interaction in the Mg(Chr)—H_2_SO_4_ system was calculated based on the reaction equations:

Mg + H_2_SO_4_ → MgSO_4_ (1)

The mass and volume of sulfuric acid for the preparation of 200 cm^3^ of a solution containing the desired amount of sulfuric acid (from 92% H_2_SO_4_ of the “chemical pure” brand) were calculated using the formulas:

M = C·M_r_·V·100/92 = C·21.30 (2)

V = m/d = C·21.30/d(3)

where: m is the mass of the desired H_2_SO_4_ (92%); C is the molar concentration; M_r_ is the molar mass; V is the volume; d is the density of H_2_SO_4_ (92%).

To study the interaction with Chrc with sulfuric acid, 10 g Chr samples were prepared, each sample was separately mixed with 118 cm^3^ with an aqueous solution of sulfuric acid containing sulfuric acid from 0 to 0.1 mol/dm^3^ H_2_SO_4_, in increments of 0.01 mol/dm^3^ H_2_SO_4_.

The process of interaction of each Chr sample (10 g) with an aqueous solution of sulfuric acid containing the desired sulfuric acid SRA was carried out in an airtight Erlenmeyer flask in a thermostat at a suspension temperature of Chr 96 °C while stirring (350 rpm) for 10 min. In an Erlenmeyer flask with a reverse refrigerator in a thermostat, preheated 118 cm^3^ (108 g) H_2_SO_4_ solution is poured 10 g chrysotile grade A-4-20, turn on the stopwatch, while stirring, stand for 10 min at 96 °C. After the expiration of time, the suspension is quantitatively transferred to a paper filter with a blue ribbon. We determine the volume, mass and pH of the filtrate. The filtration rate was calculated using the formula:

Kf = V/τ·S (Kf—mL/min·cm^2^, V—mL, τ—min, S—cm^2^).

Insoluble residue, after preliminary preparation (after drying at 105 °C, grinding and mass determination). They were subjected to IR spectroscopic, X-ray phase and chemical analyses.

The calculation of the degree of magnesium extraction according to the results of the analysis was carried out based on the content of Mg = 26.42% in the composition of the initial Chr (A-4-20) and the mass of the dry residue of the filtrate and the Mg content in the test sample, in %. The composition of 10 g of Chr contained 2.642 g or 0.1100 mol Mg (Mg_init_ = 0.2642/24 = 0.1100 mol).

The IR-Fourier spectra were captured on a Shimadzu IR Prestige-21 spectrometer (Shimadzu, Columbia, MD, USA) with the prefix of the disturbed total internal reflection miracle by Pike Technologies.

X-ray images were taken on a D8Advance (Bruker, Billerica, MA, USA) device, Cu-K_α_, tube voltage 40 kV, current 40 mA. The processing of the obtained diffractograms and the calculation of interplane distances were carried out using EVA software (DIFFRAC.EVA V4). The decoding of samples and the search for phases were carried out using the Search/match program using the PDF-2 Powder Diffractometric Database (JCDD).

The chemical analysis was performed on a JSM-6490LV device, JEOL (Tokyo, Japan), complete with INCAEnergy 350 energy dispersive microanalyzer systems (Oxford Instruments, High Wycombe, UK).

## 5. Conclusions

Investigation of the interaction of chrysotile and aqueous solutions of sulfuric acid taken in the quantitative ratio of Chr (Mg, mol):H_2_SO_4_ (mol), which, according to SRA, shows that at SRA = 0.1–0.3, the “brucite layer” undergoes interaction by reaction (2), where Chr (Mg) is the molar content of magnesium in chrysotile.

Chr (Mg) + H_2_SO_4_ → Chr [(1 − 0.7)Mg] + 0.3MgSO_4_ + 0.6H^+^ (4)

On the surface of the silicate part of chrysotile, H_3_O^+^ ions appear which form acidic silanol or silanol bonds (≡Si–O–H, =Si=(O–H)_2_) with decationated SiO_4_^2−^ surface tetrahedra. These acid groups form one-dimensional silicate chains, dimers and trimers with transverse bridges (Si–O–Si), where the angles straighten (Si–O–Si = 180°), which are recorded in the IR spectra in the range 1130–1250 cm^−1^. The bands in the IR spectra 3620–3000 cm^−1^, 1620 cm^−1^ and 1130–1250 cm^−1^ belong precisely to these chain groups with adsorbed water, which are the surface mode of acid-treated chrysotiles.

This circumstance indicates the transformation of the silicate component in the chrysotile molecule into silica, the intensity of which increases markedly with an increase in SRA H_2_SO_4_ in the solution used to leach serpentine, leading to some inhibition of the chrysotile dissolution process when using a concentration of SRA H_2_SO_4_ ˃ 0.3.

## Data Availability

The data used to support the findings of this study are included within the article.
